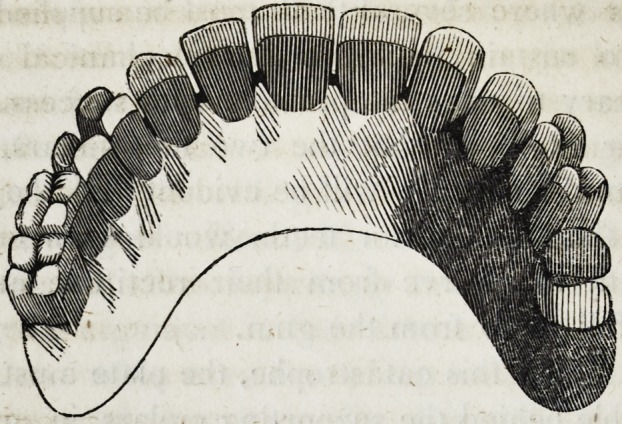# A Treatise on Mechanical Dentistry

**Published:** 1842-03

**Authors:** Solyman Brown


### ARTICLE II.

*A Treatise on Mechanical Dentistry.* By Solyman Brown, M. D., D. D. S.

(Continued from page 177.)

### CHAPTER II.

*Of the insertion of several teeth on natural roots, in cases where the number of natural roots does not equal the number of teeth to be inserted.*

37. It sometimes happens that several good roots remain in the mouth, fit to sustain artificial crowns, but that some one or more roots have been removed either from between or near the others, so that a complete arrangement cannot be effected on the principles laid down in the first chapter.

38. Let it be supposed that the crowns of the four upper incisors and the two cuspidati, are lost, and also the roots of the right cuspidatus and left lateral incisor; as in the following cut.

In this case although the four remaining roots would sustain four separate crowns independent of each other, yet there would still be two vacancies which are to be supplied; and though in the case supposed in the cut, there are back teeth which might be used to sustain a plate, with clasps, yet the patient might not consent to the use of them; and cases moreover often occur in which there are no back teeth in the mouth, leaving it imperative on the dentist to sustain the six front teeth on four roots, as in the cut.

39. Take a wax impression of the front part of the mouth, embracing at least the entire space to be supplied with teeth, as described in sections 16 and 17, from which proceed to obtain metallic casts as explained in sections 18 and 19. To that part of the tin cast representing the space to be occupied with artificial teeth, fit a plate of tin or lead rolled to the thickness of drawing paper, so that it shall cover the ends of the roots together with the space left by the two teeth that have been extracted, extending back into the mouth three-fourths of an inch, and passing behind the first bicuspides on each side of the mouth. Bring the two casts together upon the soft metallic plate, and then trim it to the following shape.

*a* is the front part covering the roots; *b* the back part extending to the roof of the mouth or palate; *c* and *d* are points of the plate which serve to retain it in its place while struck between the casts, but are afterwards cut away in case they are too much in sight; *e* and *f* are points extending behind the first bicuspides, which serve to sustain the piece steadily in its place; and especially prevent the whole apparatus from being pushed foward by the action of food, the tongue, or the antagonizing teeth.

40. Reduce the soft metallic plate to a plane surface under a planishing hammer, and employ it as a pattern by which to cut the gold plate which must be struck between the casts as explained in sec. 20. In swedging large plates which are to be much bent between the casts, care must be taken to anneal the gold plate once or twice during the operation, which is done by simply heating it to redness either under the blowpipe, or in a coal fire, care being taken that the gold be not melted by excessive heat. The following instrument will be found very useful in bending gold plates into a shape approaching that given by the casts, in order to allow the casts to be brought together properly.

41. Pierce the gold plate with small pivot holes by means of the plate punch shown in section 22, exactly over the nervous orifices in the roots, into which solder pivots of gold or platina of the size of a small knitting needle, and one-fourth of an inch in length; after which clean the plate, file it smooth, and cut off the corners *c* and *d* of the plate, if the case requires it.

The plate and pivots will now assume the following appearance.

Adjust this plate to a plaster cast which has been reserved for this purpose, in order that the artificial teeth may be ground and adjusted to the plate just as it is desired they should stand in the mouth. To this end support the teeth in their just position by means of softened beeswax placed behind them on the plate to which it may be made to adhere by warming the plate.

If the patient can be seen at this stage of the process, instead of fitting the teeth as just described, according to the best judgment of the operator, cover the plate with softened wax, which place in the mouth, and bring the ends of the teeth of the lower jaw against the wax till the mouth is shut in its natural position. The impression of the antagonizing teeth in the wax, will be a sure indication of the just position of the artificial substitutes. The wax may be cut away with a penknife as each tooth is adjusted in its place.

If, on the other hand, the operator finds it necessary to fit his teeth to their place before seeing his patient, the whole may be tried into the mouth by proper care; or if sealing-wax be used instead of beeswax, the trial will be attended with no difficulty.

42. When the mineral teeth have been set in the position required, and ground at the butts so as to fit the plate accurately, and project beyond it in order to touch and even press upon the gum, cover the enamelled faces of the teeth with a mixture of plaster and sand in equal parts reduced to a paste with water. This mixture is little liable to crack under the heat of the blowpipe, especially if secured by a few turns of fine iron wire, such as dentists frequently need in various parts of their work. This wire is little thicker than a bristle or coarse hair, and is so soft and pliable as to be little liable to break.

Remove the beeswax from behind the teeth, which may be effected with a penknife, or by the aid of warm water.

43. Each tooth may now be removed from its place, and a gold back or stud may be fitted to it as described in section 22. Care should be taken that the gold back be fitted closely not only to the tooth but to the plate, and if, by accident, the junction with the plate should be imperfect, the vacancy may be filled with gold foil carefully introduced so as not to change the position of the tooth. Over this foil the solder will follow, and even incorporate itself with the foil, uniting the whole into one solid mass, especially if a sufficient quantity of solder be employed. When each tooth has its gold back thus properly adjusted, the whole piece will have the following appearance as seen from behind.

44. When the plaster has been thoroughly dried, which may be done in an oven, provided the operator be in haste, or over a fire in a ladle, provided the degree of heat used be not sufficient to *recalcine* the plaster, the piece is ready to be soldered.

In order to perform the operation of soldering with neatness, solidity and beauty, apply the borax to the back of the tooth only, quite down to the plate, by means of a flat, thin piece of cane or soft wood. A sufficient quantity of the borax will run down upon the plate without applying any to that, and if the operator has any fear that the borax will carry the solder over too great a portion of the plate, he may apply a thin coating of whiting to all that part to which he wishes no solder to run, leaving in all cases a semicircular spot behind each tooth, which must be kept quite clean in order that the solder may run over it unobstructed. Next, apply a quantity of solder to the back of each tooth, nearly or quite equal to the weight of the back itself. This may be in one or several pieces, at the pleasure of the operator. Let the borax dry, if time will permit, lest it should displace the solder during the sudden conversion of the water into steam by too great a degree of heat.

45. Before applying the blowpipe to a piece like this, the hollow cup of charcoal mentioned in the first chapter should be prepared as follows:—

Let the operator not forget to apply the heat with caution, lest the teeth be cracked by sudden and unequal expansion, and let the heat be continued until the solder assumes the form best calculated to give strength and beauty to the work. This form which the solder should assume, may be thus represented on a single tooth when seen laterally with a section of the plate.

Should the quantity of solder on any tooth be found insufficient, more may be added during the process of soldering, and the heat again raised to the point of fusion.

In soldering pieces like this, some advantage would be gained, if the right hand of the operator could be at liberty to make use of a rod of platina of the size and length of a knitting needle, with which he may move the melted solder about on the gold plate at pleasure, in order to bring it into the desired form.

To accomplish this object, it will be necessary to use one of Hook's self-acting blowpipes, which, with an improvement made by Dr. Jahial Parmly, of Bond street, New York, will be found of great importance to the mechanical dentist.

This blowpipe, as is well known, consists of a brass globe composed of two hemispheres screwed firmly together, having an orifice at the top for the purpose of introducing alcohol, and a tube leading from the upper hemisphere to the flame of a lamp placed underneath the brass sphere. The whole may be supported on a stand as follows:—

Without entering more minutely into the details of the formation of this instrument, I will merely add, that when the globe is partly filled with alcohol, and the lamp lighted beneath it, a part of the alcohol is soon converted into vapour, which finding no vent excepting through the small tube leading to the upper hemisphere of the brass globe, the vapour or steam of the alcohol is forced from the orifice of the tube directly against the flame of the lamp by which the alcolic steam itself takes fire and forms a jet of flame of great intensity. Dr. Parmly's improvement consists in having two distinct wicks in the lamp with their appropriate tubes, so that one may act on the globe while the other is wholly diverted from it, or both may be directed upon the piece to be soldered when that may be *necessary.* With this instrument Dr. Parmly has succeeded in preserving the solder in a state of fusion for any required time, while with his platina rod he has given to the solder the form and position required, and all this without raising the temperature enough to fuse the gold plate.

When the piece in question has been properly soldered, cleaned and polished as already described, the pivots may be wound with a little raw cotton, or floss silk, before fixing the plate firmly in the mouth, in order to avoid the wearing away of the roots by the metal ; or when the cavities in the roots are very large, they may be filled with soft wood, and the pivots inserted into these wooden plugs.

*Of the insertion of a single tooth in the absence of a natural root.*

46. This is so nice and difficult an operation that I shall deem it expedient to present several examples, inasmuch as we find that many dentists who can construct a good double set—rarely ever succeed in setting a *single tooth* well.

Let us first suppose that the tooth to be supplied, is a central incisor of the upper jaw ; and that it is intended rather for show than mastication, inasmuch as the patient is unwilling to submit to an arrangement that would render the artificial substitute fit for general use.

After fitting a gold plate to the space to be occupied by the tooth, as already directed, solder a wire of fine gold or platina to each end of the plate, of sufficient length to embrace the two adjacent teeth as far as those next to them will permit, as follows ;

Such a plate with its clasps of wire, when well fitted to the gum and the two adjacent teeth, is found to sustain a tooth in its place with sufficient firmness for all purposes excepting that of masticating food; but if a tooth be desired for the performance of this latter function, another plan must be pursued.

Select two of the molares or bicuspides, one on each side of the mouth, around which clasps of gold may be adjusted, either with or without filing a passage for the clasps, as circumstances may require. To these clasps attach the extremities of a plate constructed as already described, and sustaining the tooth as follows:

As the best methods of attaching a clasp to a plate in a neat manner, are of some importance to the student, I may state that winding with fine iron wire will sometimes succeed, yet the following mode is always most efficient and exact. The clasp which in most cases should be as wide as the tooth it embraces will admit, may be secured to the gold plate while both are on the model, by joining them with beeswax, or, when necessary with sealing-wax. Then lift the whole carefully from the model, lay it on a piece of paper with the side on which the wax is downward, and pour plaster upon the upper side until both the clasps and plate are imbedded in the plaster. When the plaster is set, remove the wax, dry the plaster, apply the borax, and solder as usual.

In this as in all other cases when two pieces of gold plate are to be united with solder, if the two do not accurately meet, fill up the vacancy with gold foil before applying the borax and solder, as already directed.

This method of insertion, although more expensive than with one clasp only, has the advantage of greater firmness and durability.

The disadvantages of such a plate and clasps, are, the injury accruing by necessity to the teeth which are embraced by the clasps, and the inconvenience experienced by the tongue from the presence of a plate in the front of the mouth, which the tongue meets in articulating all the lingual sounds. Both these disadvantages, however, are generally preferred to the absence of a tooth from the front of the mouth.

In cases like the foregoing, for the purpose of preparing a metallic cast in such a manner that the gold plate struck upon it shall fit accurately to the natural teeth behind which it passes, cut away from the plaster model all the teeth nearly level with the gum, then after taking the metallic casts from this, strike the gold plate over the whole, and cut away the plate accurately with round files, where the natural teeth are to meet the plate.

47. It happens not unfrequently that, either on account of the loss of the teeth on one side of the mouth, or for some other solid reason, an attachment can be made to only one tooth, or to those on but one side of the jaw. In the former case a piece may be constructed as follows.

If the plate and spring, or clasp, are of considerable thickness and strength, this arrangement will be successful, even for purposes of mastication, but it is desirable in many cases, if practicable, to take two points of support, as follows:

In all cases the clasps embracing natural teeth for the purpose of supporting artificial ones, should be as wide as possible, in order to avoid wearing away the teeth at the neck.

48. Let us next suppose that the second bicuspis has been removed, and the dentist is required to supply its place. The object in this and all other cases, being to throw the fastenings as far back into the mouth as possible, in order to be out of sight, let the clasp embrace one side of the first molaris, and both sides of the second, thus:

This method of fixing will prove very firm, provided the strength of the materials be justly proportioned to the use to which the artificial substitute is to be applied.

In those cases where the next tooth is the only one to which attachment can be made, it must be evident that no great dependance can be placed on the firmness of the work. If however the tooth which is to sustain the piece, be either very flat like some bicuspides, or very strong and angular like some molars, the work will not fail to be of use. Take for example a first bicuspis and a first molaris.

In the former case, if the second bicuspis be very flat the clasp will be little liable to a rotary motion. In the case of a first molaris, when the second is strong and angular, the most perfect success will attend the operation. In both these cases the strength of the material together with the peculiar form of the supporting tooth, will secure the piece from being displaced by any other means than hard food which must not be allowed to come in violent contact with so slight a fixture.

*Of the insertion of several teeth on a gold plate with clasps sustained by natural teeth.*

49. As cases of this description are very various, and not less in number than the arithmetical changes that can be rung on 32 bells, I shall deem it necessary to give only a few examples as specimens of the whole: and I begin with the most common and most important case of this kind, which presents itself in supplying the two central superior incisors.

Although there can be little doubt of the superior permanence and utility of a plate of this kind attached to some of the back teeth, thus:—

Yet many American dentists and most of the English practitioners would treat the case as follows:—

Here the whole apparatus is secured in position by means of two small gold wires encompassing in part the lateral incisors. Some assistance however is rendered by the narrow neckings on that part of the plate which is adjacent to the neighboring teeth. These neckings must fit with great exactness or they cannot be endured by the tongue. Pieces like this are less liable, perhaps, than some others to affect injuriously the natural teeth: which is certainly a great argument in their favour.

50. Sometimes when even four of the incisors are to be supplied, the same principle is successfully observed, thus:—

But in almost all cases of this kind, I should prefer an attachment to some of the back teeth when practicable, as follows:

If two bicuspides are to be supplied as is not unfrequently the case, let the following be a sample:

51. In cases where several teeth must be supplied, and only a few remain to sustain them, various mechanical contrivances become necessary to solve the problem with success.

Suppose, for example, that the two first molars, are the only remaining natural teeth. It will be evident that the weight of a plate bearing the ten anterior teeth, would soon cause the two supporting teeth to swerve from their rectitude and allow the front teeth to fall away from the gum.

In order to avoid this catastrophe, the plate must be extended as far as possible behind the supporting molars, in order to find a fulcrum or sustaining point to the lever here represented by the gold plate.

These extremities of the plate may be used to sustain artificial teeth, or they may be left unoccupied, but their presence is essential to the proper construction of the piece, inasmuch as the two supporting molars must be drawn perpendicularly from their sockets before the front can fall in consequence of their motion; whereas a mere lateral declination of these supporters would produce that effect in the absence of these extremities of the plate behind the two natural molars.

52. One of the most vexatious cases falling into the hands of the dentist, is that in which a large plate must be supported by a single tooth.

If this should chance to be a large and firm molaris, the chances of success are somewhat enhanced; in which case the piece will present the following appearance.

Cases of this character are so doubtful as to the permanence of the supporting tooth, that unless it be very firm and sound it ought not to be trusted. When however, the attempt is made to construct such a piece, let the clasp be very strong and the plate thin and light. The teeth also should be as slender as possible.

53. The operator should always avoid laying plates over roots of the natural teeth remaining in the mouth. Besides the absolute certainty that these roots will prevent the perfect fit of the plate to the gum, they are liable to be agitated by the constant action of the plate upon them by which they become loose, and often require extraction, after which the plate no longer fitting the gum, becomes useless.

Although many patients are very averse to the removal of these roots, and often allege examples among their acquaintances in which plates have been laid over these roots of the natural teeth, with perfect success, cases rarely occur in which these persons cannot be persuaded to pursue a proper course of treatment, by judicious management on the part of the dentist. Sometimes he may succeed in inducing them to submit to the operation of extraction, by showing them that their money will be wastefully expended in procuring work which rests upon a foundation insufficient and precarious; at other times, by assuring them that he is wholly averse to executing work unless he can do it in what he knows to be a proper manner, by which he can do justice both to his patient and his own professional reputation. Often he may succeed in his object by persuading the patient to permit him to remove one or two only of the loosest of the roots, after which, the charm being broken, no resistance will be made to the extraction of the rest.

At any rate, if the operator consents, in any instance, to lay a plate over the stumps of teeth, he owes it to himself to impress it on the memory of his patient that he does it in opposition to his judgment and experience, and does not consent to hold himself responsible for the result. If this statement should be entered on his books, with the patient's knowledge, it might prevent future complaints, for it happens not unfrequently that a dentist in extensive practice is blamed for operations, the failure of which is the result of the obstinacy of his employer.

54. Another precaution of which the young practitioner should be early apprized, is, never to lay a plate after teeth or roots have been extracted, until sufficient time has elapsed to allow the alveolar processes to be absorbed, and the gums fully healed, so that the teeth shall not become too short by subsequent absorption of the subjacent parts.

The dentist who neglects this precaution, will find himself frequently called on to substitute longer teeth, and will even sometimes be assured that they have been always too short from the beginning.

55. To avoid these unpleasant occurrences, let him on the one hand, insist on allowing the gums sufficient time to become thoroughly healed and settled before constructing his work, and, on the other hand, let him never forget to leave the artificial teeth as *long* as circumstances will permit. In many instances, especially when the artificial substitutes are met on their cutting edges by antagonizing teeth, the latter of these injunctions cannot be obeyed, and it is in such cases particularly, that the dentist should protect himself from both blame and loss, by keeping a record of the statements which he made to the patient on the subject.

56. It is very difficult to give any definite rule as to the period required for the absorption of the alveolar processes and the through healing of the gums after the removal of teeth or roots. In some cases a week may be sufficient, if only a single root and that a short one, has been removed. A month even would be too brief a period for the majority of cases that occur in practice, and two or three months are sometimes required. Each case is modified so much by the condition of the patient's health; by the number and character of the teeth extracted; and by the state of the natural teeth remaining in the mouth, that the period required in any given set of circumstances, is better learned by observation, than taught in theory; and this is one, among many reasons, requiring a patient apprenticeship of several years under suitable tuition, in order to enter understandingly upon general practice in dental surgery.

57. The dental operator owes it to his patient who has confided in his skill and integrity, and he owes it to justice and humanity, to inform all his professional friends who wear artificial teeth on metallic plates, of the great importance of not merely putting the mouth in a healthy condition before the introduction of artificial substitutes, but of the absolute necessity of keeping the mouth and all it contains, perfectly clean afterwards.

Nothing is better established among physiological facts, than that filth lodged among the teeth, and left to ferment, decompose, and putrify in the mouth, invariably destroys, sooner or later, all the remaining natural teeth; and thus renders useless all the clasps and fastenings of which we have treated at large in this chapter.

It may indeed be true that many patients will not comply with the instructions of the dentist on this subject, as it is unquestionable, that even some females of extreme neatness in other things, allow the mouth to be by far the most filthy place in all their habitations. They scrub their hearth-stones and require the chimneys of their houses to be now and then swept, but as to their mouths, they have really no time to keep them clean. But notwithstanding all this, the dentist who introduces artificial teeth, should urge the necessity of keeping the mouth absolutely clean.

58. It is known to every dental practitioner that although there is no single and simple acid that will corrode or dissolve gold, yet there are combinations of acid and other acrid substances allowed by some persons who wear metallic plates to remain so long and so constant in their mouths, as to destroy a thick plate of gold in a very few years.

This never could occur if the mouth were washed thoroughly with water and a brush several times a day; much less, if some slightly alkaline solution, or a good dentifrice were occasionally employed.

59. In order to aid the patient as much as possible in this duty of keeping the mouth free from impurities, the dentist should in all cases, if not impracticable, so construct his work that it may be readily removed from the mouth.

The springs or clasps which have been already described, should be made sufficiently fast to sustain the plate and teeth, but at the same time capable of being slipped from the natural teeth which they embrace, at the pleasure of the wearer.

To this end it may be sometimes necessary to restore the elasticity of the metal which has been destroyed by the heat employed in soldering. This is effected by the strokes of a small hammer; and by using a small anvil called a beck-horn, it may be done with ease, especially in those parts of the clasp where the elastic spring is most required.

60. This elasticity of the clasp is most required in those cases where the neck of the tooth which sustains the plate, is considerably smaller than the crown over which the clasp must be first passed. I have sometimes been compelled to obviate this difficulty by means of an elastic gold ferule made of very thin plate and wound in concentric circles in the manner of the mainspring of a watch. This should be so constructed that when encompassing the neck of the tooth it shall embrace it firmly. Over this the clasp attached to the plate may be readily passed, if all parts be properly constructed.

[TO BE CONTINUED.]

## Figures and Tables

**Figure f1:**
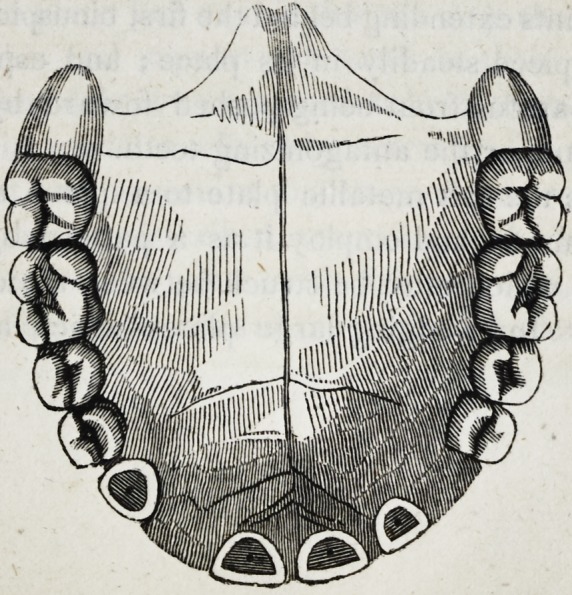


**Figure f2:**
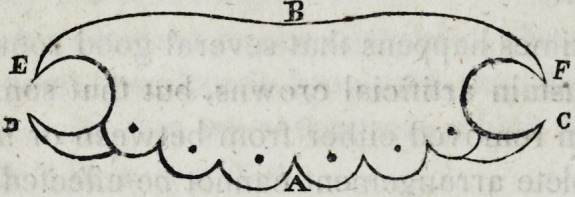


**Figure f3:**
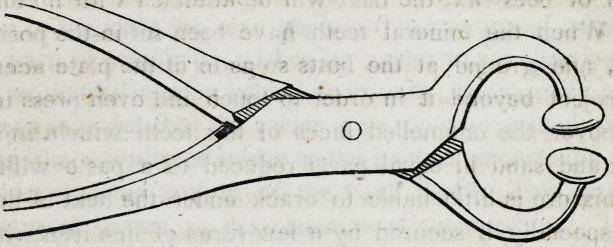


**Figure f4:**
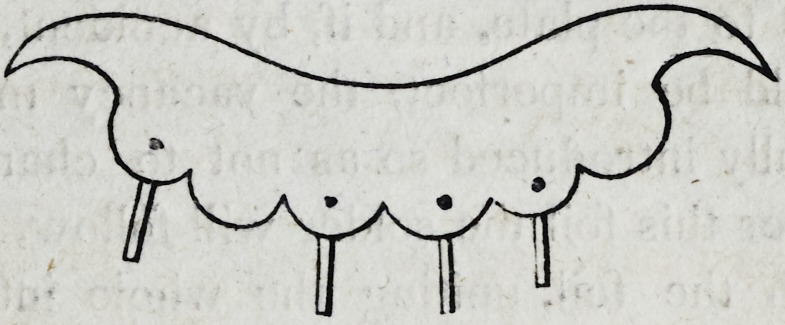


**Figure f5:**
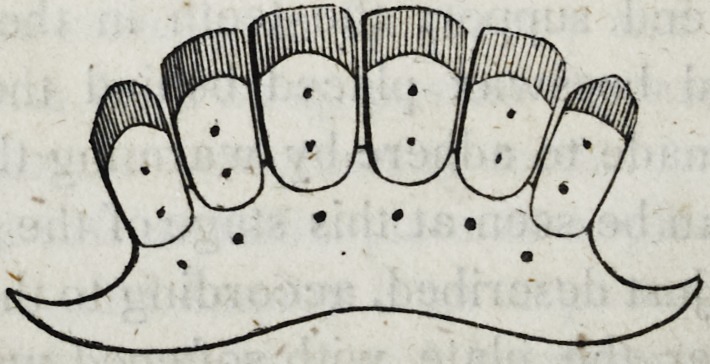


**Figure f6:**
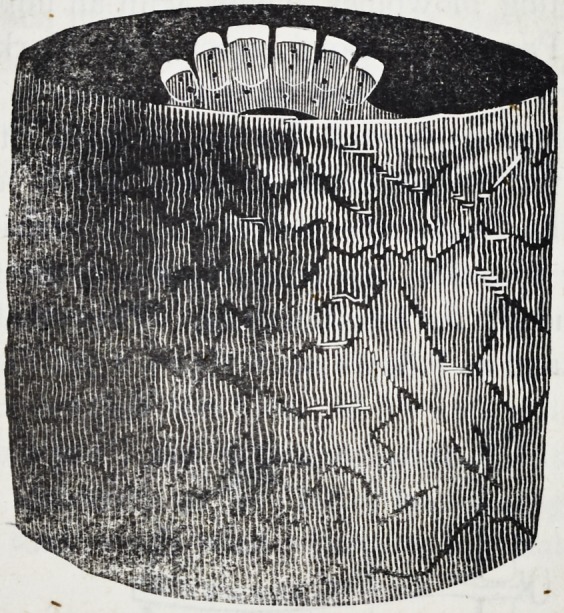


**Figure f7:**
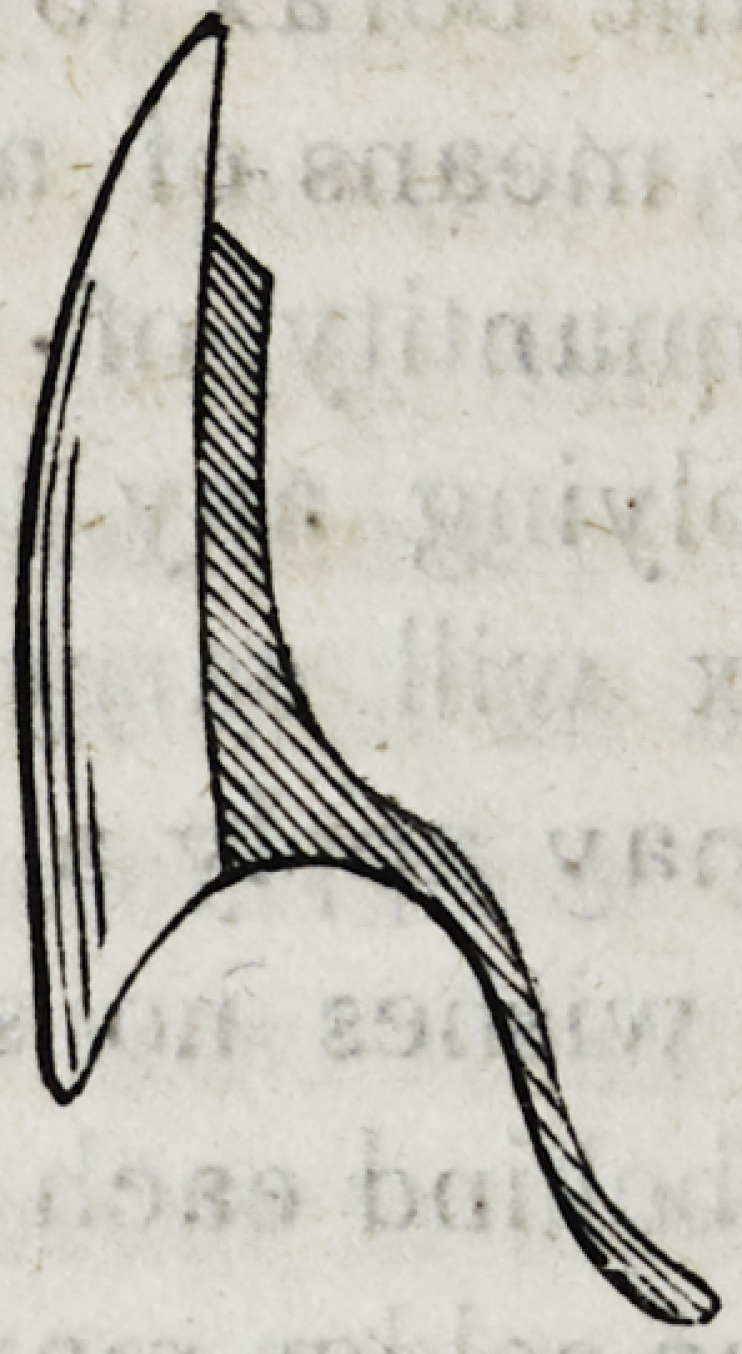


**Figure f8:**
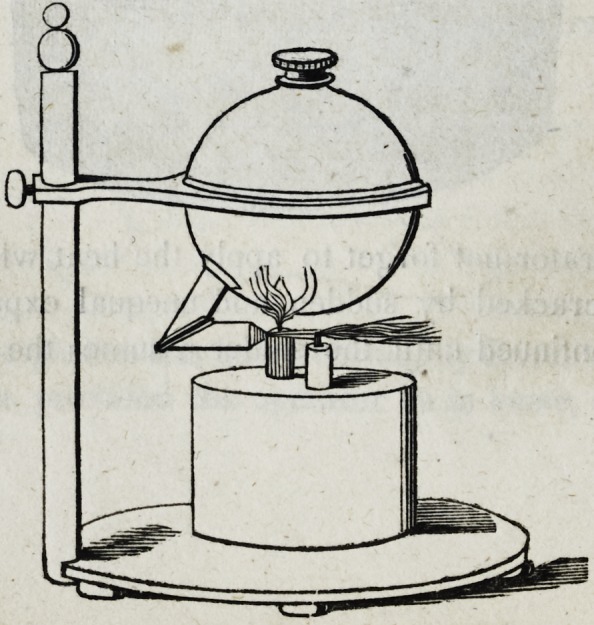


**Figure f9:**
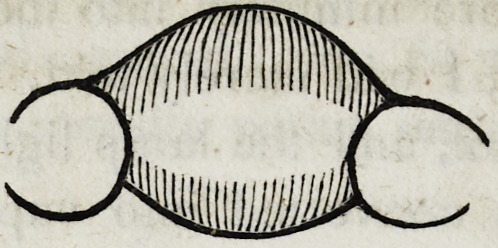


**Figure f10:**
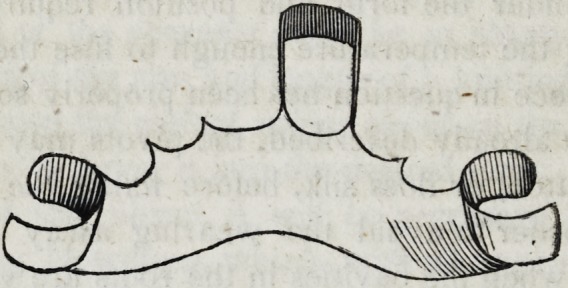


**Figure f11:**
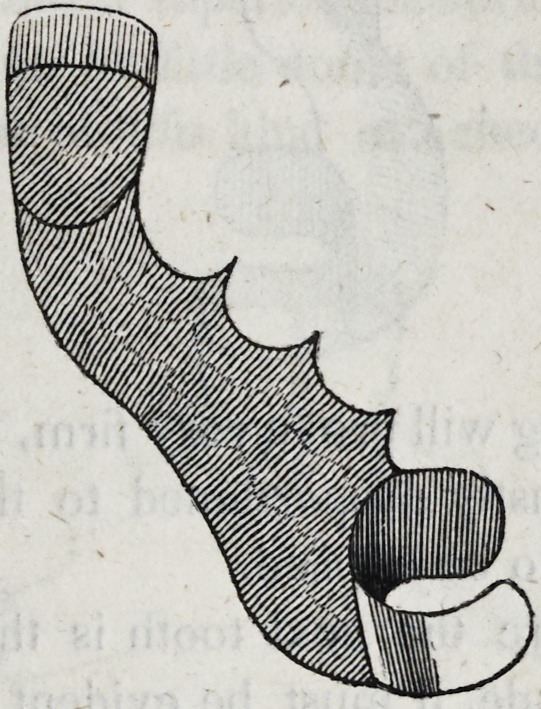


**Figure f12:**
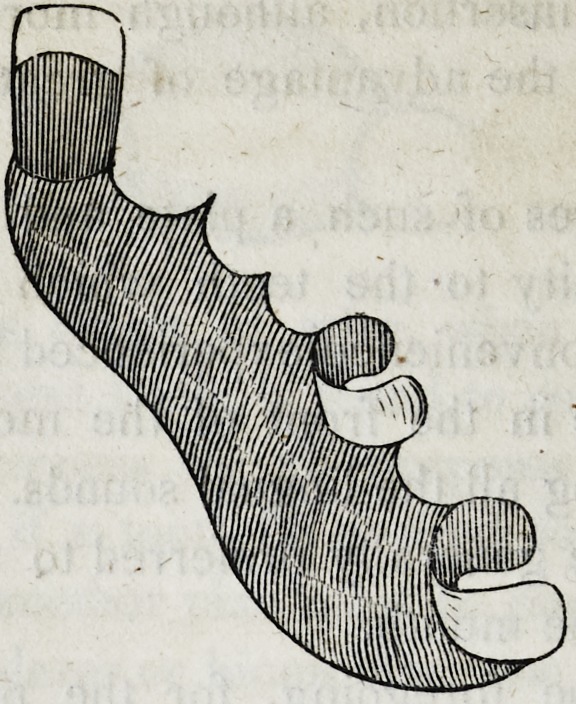


**Figure f13:**
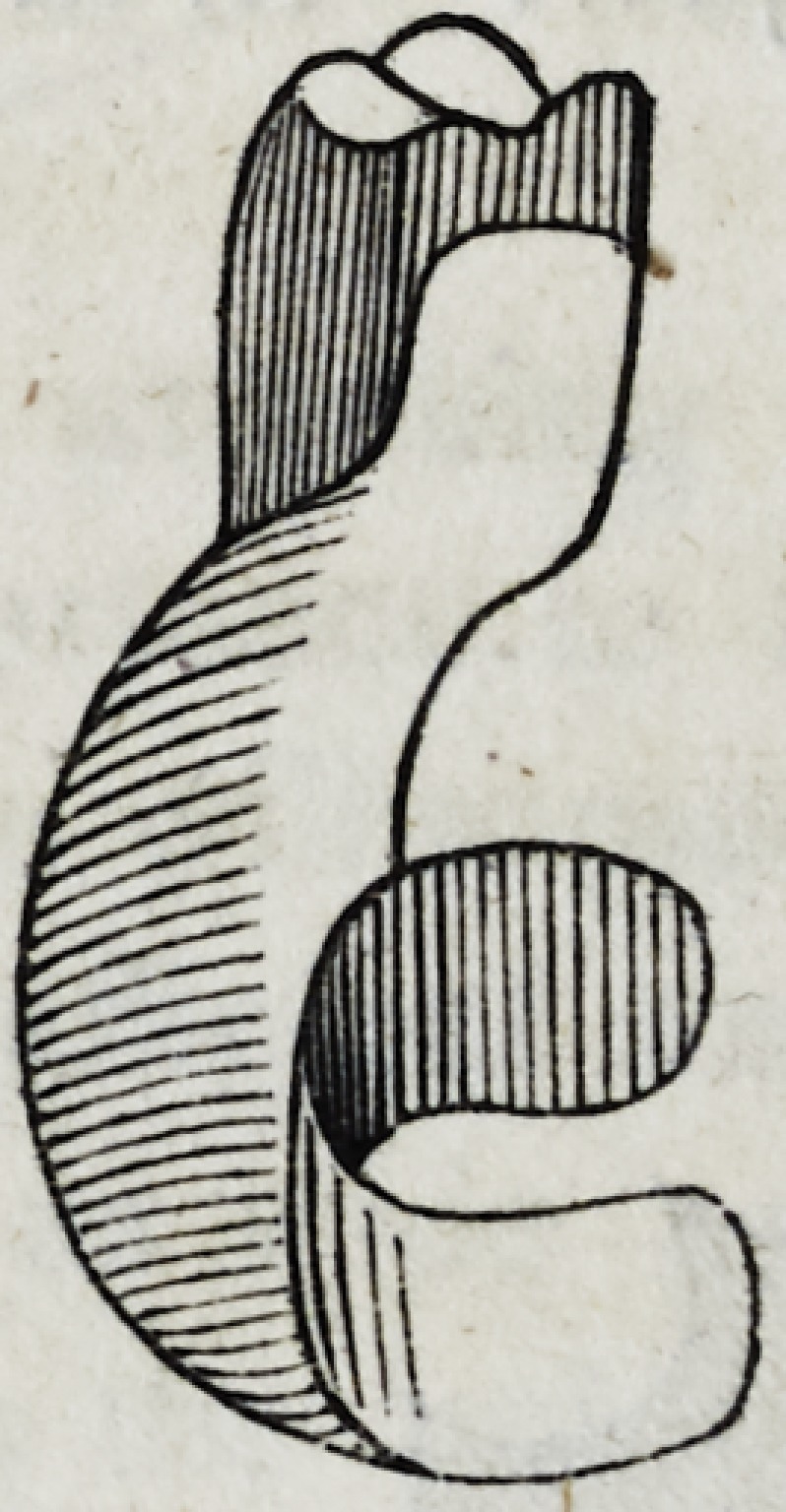


**Figure f14:**
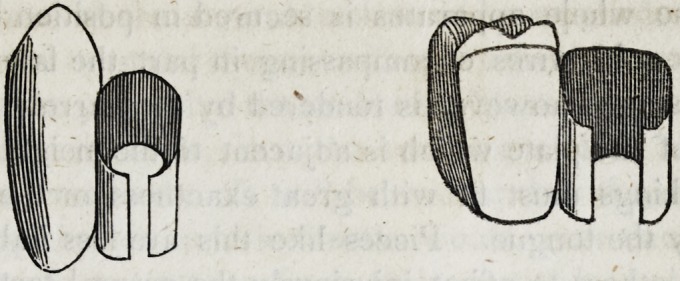


**Figure f15:**
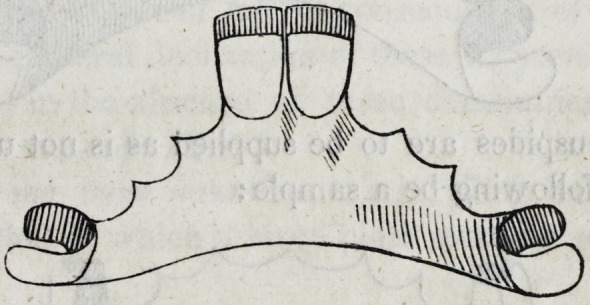


**Figure f16:**
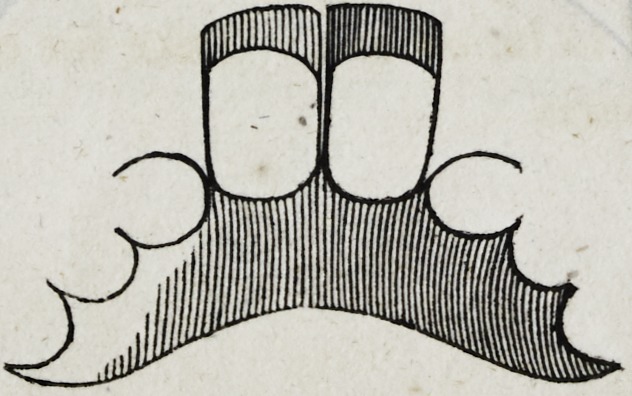


**Figure f17:**
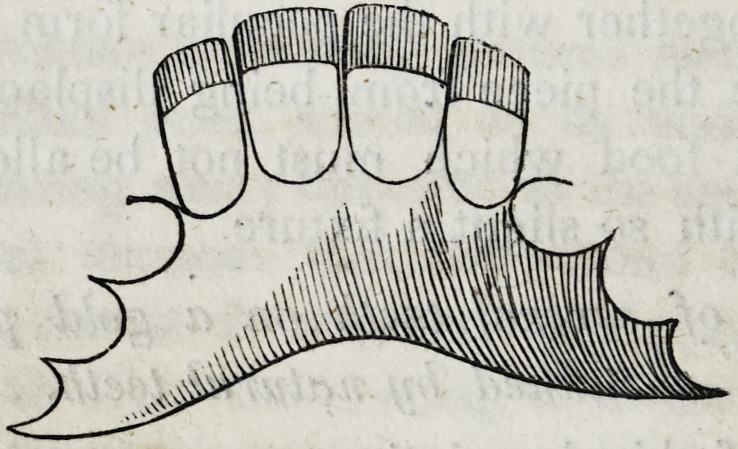


**Figure f18:**
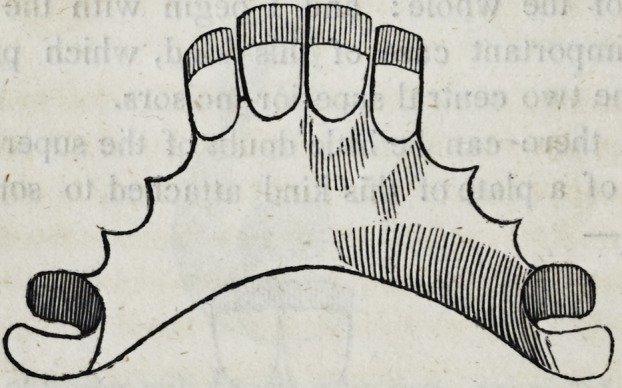


**Figure f19:**
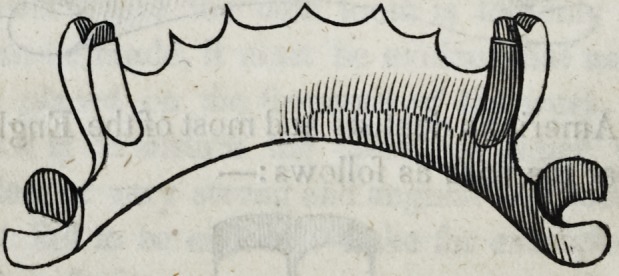


**Figure f20:**
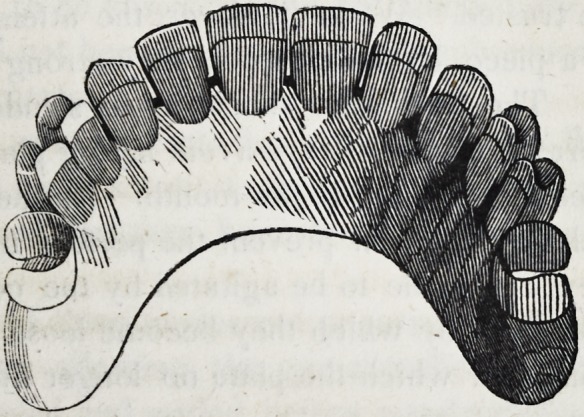


**Figure f21:**